# Tissue Microarray Analysis Applied to Bone Diagenesis

**DOI:** 10.1038/srep39987

**Published:** 2017-01-04

**Authors:** Rafael Barrios Mello, Maria Regina Regis Silva, Maria Teresa Seixas Alves, Martin Paul Evison, Marco Aurelio Guimarães, Rafaella Arrabaca Francisco, Rafael Dias Astolphi, Edna Sadayo Miazato Iwamura

**Affiliations:** 1 Escola Paulista de Medicina—Universidade Federal de São Paulo, Department of Pathology, São Paulo 04023-062, Brazil; 2 Northumbria University, Faculty of Health and Life Sciences, Newcastle Upon Tyne NE1 8ST, United Kingdom; 3 University of São Paulo Ribeirão Preto Medical School, Centre for Legal Medicine, Ribeirão Preto 14049-900, Brazil

## Abstract

Taphonomic processes affecting bone post mortem are important in forensic, archaeological and palaeontological investigations. In this study, the application of tissue microarray (TMA) analysis to a sample of femoral bone specimens from 20 exhumed individuals of known period of burial and age at death is described. TMA allows multiplexing of subsamples, permitting standardized comparative analysis of adjacent sections in 3-D and of representative cross-sections of a large number of specimens. Standard hematoxylin and eosin, periodic acid-Schiff and silver methenamine, and picrosirius red staining, and CD31 and CD34 immunohistochemistry were applied to TMA sections. Osteocyte and osteocyte lacuna counts, percent bone matrix loss, and fungal spheroid element counts could be measured and collagen fibre bundles observed in all specimens. Decalcification with 7% nitric acid proceeded more rapidly than with 0.5 M EDTA and may offer better preservation of histological and cellular structure. No endothelial cells could be detected using CD31 and CD34 immunohistochemistry. Correlation between osteocytes per lacuna and age at death may reflect reported age-related responses to microdamage. Methodological limitations and caveats, and results of the TMA analysis of post mortem diagenesis in bone are discussed, and implications for DNA survival and recovery considered.

Bone undergoes a range of changes in the burial environment that are of forensic, archaeological and palaeontological interest. Analysis of exhumed bone may allow the mechanism of interment or disposal, burial location, time since death and time since burial to be established, and possible secondary interments to be identified^1^,^2^,^3^,^4^,^5^. Forensic, archaeological and palaeontological investigations of bone taphonomy have addressed macroscopic, microscopic, physico-chemical and molecular processes occurring post mortem^6^,^7^,^8^,^9^,^10^,^11^,^12^,^13^,^14^,^15^.

In forensic human identification, osteological analysis and DNA profiling are each of fundamental importance and understanding of post mortem changes or diagenesis can assist in ensuring investigative procedures can accommodate both^16^,^17^,^18^,^19^,^20^,^21^.

Bone diagenesis in soil is characterized by destruction of histological integrity, alteration in bone porosity and mineral crystallinity, and loss of collagen^6^,^7^,^8^,^9^. Collagen loss can be the result of enzymatic hydrolysis promoted by collagenase activity, creating pathways that facilitate microbial invasion^10^,^11^. Microbial attack in specific areas results focal microscopic destruction, during which collagen loss follows bone demineralization, leading to reduction in bone strength^12^. The extent of these changes can vary dramatically depending on the time and conditions of burial. They are especially influenced by factors such as humidity, pH and temperature: while physico-chemical deterioration is accelerated by extreme pH or high temperatures^13^,^14^,^15^, microbial activity is optimized in conditions close to neutral pH.

This study describes the application of tissue microarray (TMA) analysis to the investigation of post mortem diagenesis in exhumed human bone. TMA^22^ analysis is a method in which small cores are precisely extracted from conventional paraffin-embedded specimens and inserted into a fresh block so that large numbers of subspecimens can be analyzed together in a single multiplex. Cores from the donor block may be selected to give a representative cross-section of an original specimen. These cores, which may vary from 0.6 to 2.0 mm in diameter, are extracted with a hollow needle coupled to a precision support and then mounted in a recipient block. Up to a thousand specimens may be subsampled and multiplexed in this way. If, for example, slides are cut to a thickness of 5 μm and each of 40 sections is subsampled at the same point, there will be 200 μm between the first subsample—the entry level—and the fortieth subsample—the deep level, representing a vertical cross-section of the original block. Hence, a TMA provides a substantially more informative representation of the donor tissue specimen than the original single cut. It permits 3-D visualization of adjacent histological sections, simultaneous investigation of large numbers of samples and representative subsamples, standardization of reactions and ready comparative interpretation of results, economy of reagents and time, and collaborative use of the TMA blocks in multi-centre studies. The multiplexed histological sections in a TMA are amenable to analysis in a range of applications using a variety of histological and immunohistochemical techniques^23^,^24^,^25^,^26^,^27^,^28^,^29^,^30^,^31^,^32^. Immunohistochemistry, while routine in diagnostic pathology, is being used increasingly in forensic science, such as in investigations of post mortem wounds^33^, forensic toxicology^34^ and post mortem medical imaging^35^.

The exhumed human bone sample chosen for the investigation consisted of 20 specimens of femoral diaphysis collected following interments for known periods of burial of between 6 and 15 years, intermediate between fresh or recently-interred bone of a kind that might be encountered in forensic investigations and material of older forensic, historical or archaeological age.

Sections of each specimen were fixed in formalin and decalcified, prior to embedding in paraffin blocks for TMA preparation. Bone is formed of organic and inorganic components and has a complex histological structure, which present methodological challenges in TMA preparation. In order to assess parameters for decalcification, two different agents were used—7% nitric acid and 0.5 M EDTA—and the resultant bone sections mounted in separate multiplex blocks in order to permit their relative utility to be compared.

The use of existing TMA protocols validated on fresh specimens permitted their application to post mortem bone to be evaluated. Both standard histochemical and immunohistochemical staining methods were applied in the analysis. Hematoxylin and eosin (H&E)—widely used for differentiation of basophilic and acidophilic components of various tissues—was used to score osteocyte and lacuna preservation and measure bone matrix loss. Periodic acid-Schiff (PAS) and Silver Methenamine (SM) were used for quantification of fungal spheroids and Picrosirius red was used for detection of collagen fibers I and III. Anti-CD31 and anti-CD34 were used in an immunohistochemical assessment of endothelial cell survival.

Modern post-operative femoral diaphysis from routine autopsy and post mortem soft tissue sections of liver were used to offer controls with which the results of decalcification and TMA assembly and analysis in the post mortem bone sample could be robustly compared.

The application of TMA to post mortem bone is demonstrated, as is its utility in the measurement of variables related to bone diagenesis. Descriptive statistics are presented and an assessment of bivariate correlations between specimen and procedural parameters offered. Methodological challenges, caveats, and potential for the use of TMA in the investigation of taphonomy in bone are discussed in relation to the findings. The implications for DNA survival and recovery are considered.

## Results

### Sample attributes

The period of burial (yr.), age at death (yr.), cortical surface color, medullary surface color, dimensions (cm.), weight (g.) and decalcification time (days) of each specimen are listed in Table 1a and b for the two decalcification agents, 7% nitric acid and 0.5 M EDTA, respectively.

The period of burial of the exhumed bones irrespective of decalcification method ranged from 6 to 15 yr. with a mean of 9.87 ± 1.79 yr., and the age at death—where known—ranged from 22 to 75 yr., with a mean of 50.0 ± 14.70 yr. There was no significant difference in the mean period of burial between the groups decalcified using 7% nitric acid and 0.5 M EDTA (p > 0.05).

Means and standard deviations for the volumes of the exhumed and control bone specimens in the groups decalcified in 7% nitric acid and 0.5 M EDTA, respectively, are given in Table 2. There was no significant difference in the volume of exhumed (p = 0.136) or control (p = 0.277) bone specimens, whether decalcified with 7% nitric acid or 0.5 M EDTA. There was no significant difference in volume between exhumed and control bone specimens decalcified with 0.5 M EDTA (p = 0.987), but a significant difference in volume was detected between exhumed and control bone specimens decalcified with 7% nitric acid (p = 0.036).

### Decalcification parameters

Means and standard deviations for the decalcification time of the exhumed and control bone specimens in the groups decalcified in 7% nitric acid or 0.5 M EDTA are given in Table 2. Mean decalcification time was significantly higher (p < 0.001) in specimens decalcified using EDTA (42.89 ± 10.18 days) compared with 7% nitric acid (5.55 ± 2.38 days), but 6 of the 11 specimens were recorded as decalcified after 4 days, potentially skewing some comparisons.

Coefficients of correlation between decalcification time and specimen attributes for the two groups are given in Table 3. In the group decalcified with 7% nitric acid, low coefficients of correlation were detected between decalcification time and period of burial (R^2^ = 0.0884), age at death (R^2^ = 0.1497), volume (R^2^ = 0.1386), weight (R^2^ = 0.2593), density (R^2^ = 0.0473), length (R^2^ = 0.0229), area of largest side (R^2^ = 0.2010) and surface area (R^2^ = 0.1311). In the group decalcified with 0.5 M EDTA, low coefficients of correlation were also detected between decalcification and age at death (R^2^ = 0.1497), volume (R^2^ = 0.1292), weight (R^2^ = 0.0332) and surface area (R^2^ = 0.1940). Higher coefficients were detected between decalcification and period of burial (R^2^ = 0.5893), density (R^2^ = 0.3155), length (R^2^ = 0.4476) and area of largest side (R^2^ = 0.3921).

**Figure 1:**
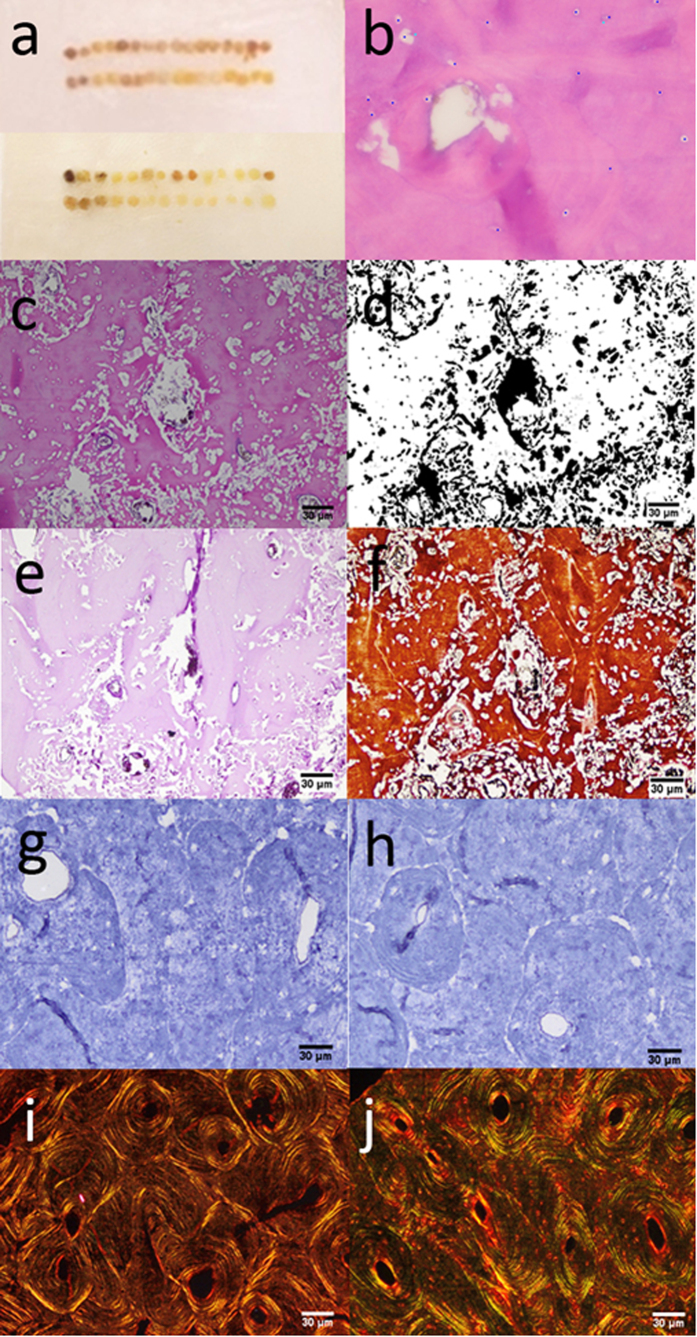
(**a**) Upper: Tissue microarray TMA-NA containing eleven exhumed and five control bone subsamples in duplicate, with a positive control (liver) for immunohistochemical analysis. Lower: Tissue microarray TMA-EA containing nine exhumed and four control bone subsamples, with a positive control (liver) for immunohistochemical analysis; (**b**) specimen 012 in TMA-NA showing osteocyte hot-spot location, ×400; (**c**) specimen 030 in TMA-EA with (**d**) same field illustrating identification of bone matrix loss in ImageJ, ×100; (**e**) specimen 030 in TMA-EA stained with PAS, ×100; (**f**) specimen 030 in TMA-EA stained with SM, ×100; (**g**) specimen 005 in TMA-NA immunohistochemically stained with anti-CD31, ×100; (**h**) specimen 005 in TMA-NA immunohistochemically stained with anti-CD34, ×100; (**i**) specimen 005 in TMA-NA stained with Picrosirius red, ×100; (**j**) control 019 in TMA-NA stained with Picrosirius red, ×100.

In preparation of the TMA blocks (see Fig. 1a), sample loss accrued at ~17 percent of cores subsampled.

### Lacuna and osteocyte counts

An example of an H&E stained TMA subsample is given in Fig. 1b. The total number of lacunae and preserved osteocytes counted in six fields in each specimen are given in Table 1 for each of the two decalcification agents. Means and standard deviations for osteocyte counts in exhumed and control samples decalcified using each agent are given in Table 2. There was no significant difference in the mean osteocyte count in the specimens decalcified with 7% nitric acid or 0.5 M EDTA, in either the exhumed bone (p = 0.377) or post-operative bone control (p = 0.248) samples. The mean osteocyte count was significantly higher in the control than in the exhumed bone samples, whether decalcified with 7% nitric acid (p < 0.001) or 0.5 M EDTA (p = 0.006).

**Figure 2:**
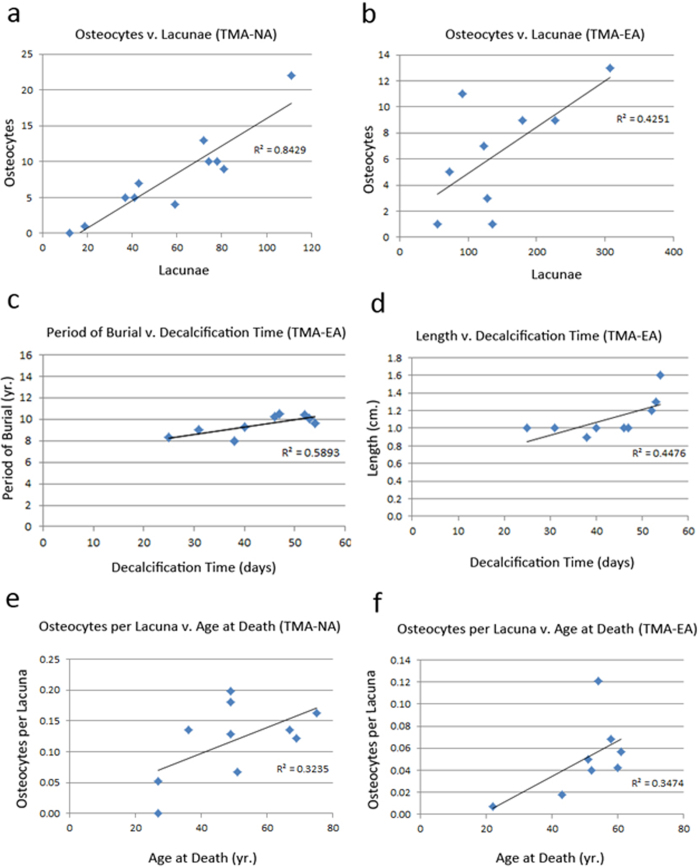
Charts showing plots and coefficients of correlation (R^2^) for selected variables: (**a**) Osteoctye count v. lacuna count (TMA-NA), (**b**) osteocyte count v. lacuna count (TMA-EA), (**c**) period of burial v. decalcification time (TMA-EA), (**d**) length v. decalcification time (TMA-EA), (**e**) osteocytes per lacuna v. age at death (TMA-NA), (**f**) osteocytes per lacuna v. age at death (TMA-EA).

High coefficients were detected between lacuna count and osteocyte count in both the 7% nitric acid (R^2^ = 0.8429) and 0.5 M EDTA (R^2^ = 0.4251) decalcified groups (see Fig. 2). Coefficients of correlation between period of burial and age at death, bone section length and osteocytes per lacuna are given in Table 4. Moderate coefficients were detected between the number of osteocytes per lacuna by age at death in both the 7% nitric acid (R^2^ = 0.3235) and 0.5 M EDTA (R^2^ = 0.3474) decalcified groups.

### Percent bone matrix area loss

Measurement of bone matrix area loss using ImageJ is shown in Fig. 1c and d. Percent loss of bone matrix area in each specimen is given in Table 1 for each of the two decalcification agents. Means and standard deviations for the percent bone matrix area loss in the exhumed and control samples for the two agents are given in Table 2. No significant difference in mean percent bone loss was observed between specimens decalcified with 7% nitric acid or 0.5 MEDTA within either the exhumed bone (p = 0.955) or post-operative bone control (p = 0.258) samples. A significantly higher mean percent bone loss was detected in the 7% nitric acid decalcified group compared with the 0.5 M EDTA decalcified group within the exhumed bone sample (p < 0.001), but no significant difference was observed in the controls (p = 0.091).

Coefficients of correlation measured between period of burial and age at death, bone section length, osteocytes per lacuna and percent bone area loss are given in Table 4. Coefficients of correlation with percent bone loss are generally low.

### Fungal spheroid element ratios

Examples of PAS and SM stained histological sections used in measuring fungal spheroid element ratios are given in Fig. 1e and f. Fungal spheroid element ratios in each specimen are given in Table 1 for each of the two decalcification agents. Means and standard deviations for the fungal spheroid element ratios in the exhumed and control samples decalcified using each agent are given in Table 2.

Coefficients of correlation measured between fungal spheroid element ratios and percent bone loss, bone section length, lacuna count, osteocyte count, osteocytes per lacuna, period of burial and age at death are given in Table 4. The coefficients of correlation with fungal spheroid element ratio detected are generally low, with the exception of that with age at death in the group decalcified with 7% nitric acid (R^2^ = 0.4840).

### Detection of preserved bone collagen fibres

Picro-sirius Red staining indicated both thick and thin collagen bundles were well preserved in all exhumed bone specimens included in the study (see Fig. 1g and h). Greater accuracy may be achieved by quantification of preserved collagen fibres and the application of other histological stains such as Silver Reticulin and anti-Glycophorin by immunohistochemistry in order to assess for the presence of red blood cells and reticular fibers, respectively.

### Detection of preserved endothelial cells

There was no expression of CD31 and CD34 markers on any of the exhumed bone samples. Expression of these markers in the post-operative bone and liver controls and were observed, however (see Fig. 1i and j).

## Discussion

Two key obstacles to TMA analysis are sample loss in preparation of the multiplex and insufficiency of recognizable and representative features detectable in the microarrayed tissues. The results indicate an average sample loss of 17%, which corresponds with values of below 20% reported in the literature^36^,^37^,^38^. Cores from at least 25 histological bone sections were subsampled and successfully embedded in each TMA block. Osteocytes were observed preserved within lacunae in 95% of specimens and well preserved thick and thin collagen bundles were observed in all specimens. No endothelial cells could be detected using CD31 and CD34 immunohistochemistry, however.

Matrix loss—due to chemical or bioerosion—and fungal invasion are fundamental features of post mortem diagenesis in bone^10^,^11^,^12^, and both were observed in this study. The provenance of fungal spheroid elements was not clear, however, and may not reflect direct fungal attack on bone.

In preserved areas unaffected by bone matrix loss, osteocyte lacunae can normally be distinguished from voids attributable to diagenesis, even in decalcified sections. They are located in compact bone and their size and morphology retains the appearance of osteocytes located in concentric lamellae surrounding the osteon (see Fig. 1b). Morphological comparison with post-operative bone controls offered a robust means of assisting in the correct identification of osteocyte lacunae by size, shape and location (see Fig. 1). High coefficients of correlation between lacuna count and osteocyte count in both 7% nitric acid (R^2^ = 0.8429) and 0.5 M EDTA (R^2^ = 0.4251) decalcified groups (see Fig. 2a and b) indicate the findings reflect the consistent detection of osteocytes within lacunae—not all of which would be expected to retain these cells as a consequence of degradation and fragmentation of nuclei.

The choice of decalcification agent appears to have a significant effect on the utility of the method (see Table 2). Decalcification using 7% nitric acid (5.55 ± 12.6 days) was substantially more rapid in comparison with 0.5 M EDTA (42.9 ± 10.2 days) and mean percent bone matrix area loss was slightly, but significantly (p < 0.001) lower (5.85 ± 3.17, compared with 6.02 ± 0.44 in the 0.5 M EDTA decalcified group). Mean osteocyte count was greater in the 7% nitric acid decalcified group (7.82 ± 6.15 versus 7.25 ± 4.06), although this difference was not statistically significant.

While an exact distinction between fungal spores and red blood cells cannot be made by morphological analysis using PAS and SM, the presence of fungal spheroid elements in 100% of PAS and 95% of SM stained subsamples and their absence from all controls offers consistent evidence of fungal intrusion.

High standard deviation values in lacuna and osteocyte counts, percent bone matrix area loss and fungal spheroid element ratios observed (Table 2) indicate histomorphological variation and post mortem diagenetic changes are not uniform even in a restricted area, and can be detected three-dimensionally using TMA analysis of serial sections with minimal sample loss in a large sample. Because of the inherent uniformity in TMA across subsamples, processing effects are discarded as an explanation for this variation, which is attributed to internal differences in the conditions of diagenesis between and within specimens. The potential for direct 3-D analysis of adjacent sections by different means is illustrated in Fig. 1c–f.

The cemetery latosoil is composed of iron oxide^39^, which can promote post mortem oxidation and degradation of the bone matrix and cellular structures in the burials, exacerbated by the acid soil, and warm and humid climate. Nevertheless, considerable variation in the range of preservation and degradation—as indicated by osteocyte count, percent bone matrix area loss, and fungal spheroid element ratio measured irrespective of the decalcification method—reinforces the perception that, regardless of the general physico-chemical conditions of the latosoil and climate, variations within each burial site significantly influence diagenetic processes. Such macrovariations, mesovariations or microvariations in the burial environment may be attributable to the incidence of sunlight and shade—which affect subsurface soil moisture and ecology, the precise depth of the burial, the availability of water, and the immediate pH, mineralogy and organic content of the soil^40^ and may have affected the cortical and medullary surface colours recorded for each exhumed bone specimen. Bone diagenesis is understood to be affected significantly by early post mortem anthropogenic factors^41^,^42^,^43^, but is not known to be associated with the external appearance of the bone. Specimen 004—an internal comparative control from another burial location in São Paulo state—did not show any distinctiveness in the results, suggesting microenvironmental parameters may be shared. A micro-analysis of the environmental variables could assist in evaluating these hypotheses.

Specimens of the femoral diaphysis are typically chosen for DNA analysis of the skeleton as they possess a cortex within which osteocytes remain protected between concentric layers of collagen and hydroxyapatite. Bones with thin cortical layers and larger portions of trabecular tissue have a more open structure^16^, which promotes microbial degradation of cellular material. Understanding of post mortem DNA preservation and diagenesis is important in forensic and archaeological—or ‘ancient DNA’—analysis as it may allow sampling from precious specimens to be optimized, avoiding inconsistencies and reanalysis, and for extraction and purification methods to be tailored to target sites of DNA preservation and to accommodate variables in post mortem bone chemistry. Among a number of diagenetic studies^16^,^17^,^18^,^19^,^20^,^21^, Misner^16^ found that mitochondrial DNA (mtDNA) was more frequently recovered from the femur and fibula (79.3%), ribs (63.6%) and pelvis (36%), and Campos *et al*.^17^ found there was a reduction in the amount of DNA in the first year after the death attributable to microbial attack regardless of environmental physical and chemical characteristics, and in subsequent years the amount stabilizes. Mundorff *et al*.^44^, however, found that cancellous bones such as the phalanges, patellae and tarsals yielded 16-locus forensic DNA profiles more successfully than the dense cortical bones commonly favoured. The teeth and, most recently, the petrous portion of the temporal bone have been shown to be useful sources of DNA post mortem^45^,^46^.

EDTA is commonly used in decalcification as part of DNA extraction protocols. EDTA incorporates bone calcium slowly as divalent bonds of oxygen enclose it in a heterocyclic chain. This reaction is called chelation and the resulting product calcium chelate. The chemical reaction continues until saturation of the chelating solution. Thus, EDTA decalcification is a self-limiting reaction requiring constant renovation^47^. Chelation of divalent cations is important in DNA extraction as these molecules may influence the polymerase chain reaction (PCR) used in downstream profiling. The relatively poor utility of EDTA evident in this study may be a reflection of its mode of action, however, which will be influenced by the hydroxyapatite matrix of the bone and its propensity to form new crystal structures that may impede decalcification^48^,^49^. A higher coefficient of correlation (R^2^ = 0.5893) detected between decalcification time and period of burial (Fig. 2c) may reflect the influence of soil chemistry on this process. The coefficient of correlation between period of burial and length was low (R^2^ = 0.1306) and the correlation (Fig. 2d) between decalcification time and bone section length (R^2^ = 0.4476) may indicate that decalcification is retarded if the specimen is sectioned to present larger surfaces in the predominant direction of the Haversian systems.

Understanding of patterns of bone matrix loss, survival and histological and molecular preservation may support greater understanding of mechanisms and localities of elevated DNA survival, which varies between skeletal elements. According to some authors, degradation of cellular components of the bone causes the release of DNA fragments of various sizes that could bind to collagen fibrils or enter into microscopic pores in the bone. These fragments would be available to mix within a saturated solution of calcium and phosphate ions, and be adsorbed by hydroxyapatite crystals that may be precipitated again in other parts of the bone tissue^19^. Prufer *et al*.^50^ reported DNA fragments from bones typically have a size of 60–150 base pairs—equivalent to a fragment size of 22–54 nm. This size is consistent with the size of hydroxyapatite crystals present in bone of 2–5 × 15–55 × 5–25 nm. It is therefore possible that DNA may adhere to the bone matrix during bone formation and remodeling, and following apoptosis or the actual death of the individual. Campos *et al*.^17^ investigated which of the components of the bone matrix—hydroxyapatite or collagen—yielded the greatest amount of DNA. The outcome of the study indicated that most of the DNA is recovered from the mineral portion of the matrix, corroborating the above propositions. Most recently, Dangaard *et al*.^51^ proposed an enzymatic pre-digestion step be incorporated in EDTA mediated decalcification targeted at DNA recovery from bones and teeth.

Osteocyte lacuna density^52^ has been reported to change in an age related pattern. No correlation between lacuna count and age at death was evident in the 7% nitric acid decalcified group (R^2^ = 0.0057), but a correlation (R^2^ = 0.3061) was evident in the 0.5 M EDTA decalcified group. A consistent correlation with the number of osteocytes per lacuna was detected in both the 7% nitric acid (R^2^ = 0.3235) and 0.5 M EDTA (R^2^ = 0.3474) decalcified groups (see Fig. 2e and f), however. These findings are interesting in the context of the observation that osteocyte density may be related to microdamage anticipated to be more frequent in older individuals.

While the study demonstrates TMA can be applied to post mortem bone and to the measurement of parameters relevant to post mortem diagenesis, certain limitations and caveats must be taken into account. Decalcification of the bone inevitably affects the bone matrix and may interfere with the attribution of changes to post mortem processes. Comparison with undecalcified controls may allow this issue to be resolved, but decalcification is implicit to TMA and may represent an inherent limitation to the method. Application of TMA to highly degraded bone may be unfeasible. Differences in the pattern of bone matrix loss ascribed to the decalcification methods used could reflect underlying systematic differences or bias between the two sets of samples. The effects of decalcification and TMA processing could be assessed via the post-operative bone fragment comparative controls (Tables 1a,b and 2), however. Only lacuna count is substantially elevated in the 0.5 M EDTA decalcified group and a higher correlation (see Fig. 2a and b) between osteocyte and lacuna counts in the 7% nitric acid decalcified group may indicate identification of osteocytes within lacunae may be more reliable when is this agent is used. There is no apparent difference in the fungal spheroid element ratios detected using either decalcification method.

Using the approach adopted in this instance, it was not possible to distinguish between erosion arising from chemical, bacterial or fungal attack^41^,^53^,^54^,^55^, to assess transverse patterns—such as those affecting the periosteal and sub-periosteal or endosteal and sub-endosteal zones^41^,^43^,^56^, or to determine the mechanism of attack and detailed aetiology of bone matrix loss^41^,^53^,^57^,^58^,^59^. Bioerosive tunneling can be observed in TMA sections (see Fig. 1b), but decalcification would—for example—remove the hypermineralized barrier defining bacterial tunnels, impeding their identification. Bell *et al*.^5^ noted tunneling forming around osteocyte lacunae, from which diagenetic lesions may emanate^60^. Histological analysis of other sections in this sample undertaken using standard (non-TMA) methods demonstrated Type 2 Weld tunneling^61^. These may also be evident in TMA sections (see Fig. 1c, for example), but this is not clear. These questions could be explored further using TMA applied to different subsamples using different histological techniques, however. Finally, the possibility that apparent correlations between variables are due to chance might be addressed via analysis of a larger set of subsamples, permitting multivariate analysis of correlations and their statistical significance.

## Conclusion

The study demonstrates the application of TMA to post mortem bone specimens and the measurement of standardised immunohistochemical and histological parameters relevant to the investigation of taphonomic processes. Lacunar osteocyte counts, bone matrix loss and fungal spheroid element counts were measured in subsamples offering broad representation of the original tissue section and permitting direct quantification of bone matrix loss. The effect of decalcification on the bone matrix may interfere with attribution to post mortem processes, however, and this may represent an inherent challenge of TMA. The presence of thin and thick collagen fibre bundles was demonstrated in all specimens, but no endothelial cells could be detected by immunohistochemistry.

Initial findings indicate that decalcification using 7% nitric acid proceeded far more rapidly than with 0.5 M EDTA, and histological and cellular structure may be better preserved using the former. Although the subsamples cannot be proven to be wholly representative, they do offer consistent analysis of a common bone section in a large sample arising from the same burial location, interred and exhumed of over a comparable time period. A wide range of variation in taphonomic parameters measured suggests that burial microenvironment may affect diagenesis substantially. Correlations observed between lacunae housing osteocytes and age at death may relate to proposed models for response to microdamage. The significance of apparent correlations may be verified by applying the TMA methods demonstrated to more extensive representative microarrays generated from larger suites of samples. Similarly, limitations related to discrimination between different forms of chemical and bioerosion using TMA may be addressed via further methodological development.

The study shows that TMA can be successfully applied to post mortem bone, efficiently permitting 3-D visualization of histological sections, simultaneous study of large numbers of samples and representative subsamples, standardization of reactions and ready comparative interpretation of results. Further research applying TMA to the investigation of the microstructure of bioerosion^57^,^58^,^59^,^60^,^61^,^62^ and indices of bone integrity, as well direct DNA profiling of the histological sections^63^ or cores may advance understanding of preservation and diagenesis in this and other samples.

## Methods

### Ethical approval

The remains were donated by the deceased’s families for anthropological research at the Medico-Legal Centre, Department of Pathology, Ribeirão Preto School of Medicine—University of São Paulo (FMRP-USP). The study was approved by the Ethics Committee of the Escola Paulista de Medicina of the Federal University of São Paulo (EPM/UNIFESP) under Plataforma Brasil number 534 593 -CAAE 27202814.0.3001.5440 and is complaint with the Informed Consent and Agreement Committee (Resolution 196/96 of the National Health Council of Brazil) in that the human remains to be analyzed were in an ossuary of the cemetery. All samples were derived from male or female individuals over 18 years old. No personally identifiable information was used. The project also has the approval of the Economic Development Company of Ribeirão Preto (CODERP), the company that administers the cemetery from which the bones were exhumed.

### Exhumed bone and control sample donor block preparation

Specimens of compact bone were collected from the femoral mid-shaft of the skeletonized remains of 19 individuals exhumed during 2012 and 2013 following redevelopment of part of a public cemetery in Ribeirão Preto SP, Brazil. The cemetery soil is characterized as slightly acid—pH 6.4—red latosoil composed mostly of clay, water, acric latosoil, carbon and iron^39^. The climate is tropical semi-humid, with a rainy season in the late autumn and a dry winter. Average temperatures are above 18 °C in all months of the year, with an annual average of 21.9 °C and rainfall of about 1500 mm per annum. The interments had taken place between 2000 and 2006, in wooden coffins buried at about 1.5 m. below the ground surface. A further exhumed specimen—004—from a location in São Paulo, SP, was included as an internal comparative control.

A modern control sample of specimens of femoral mid-shaft from a further 9 individuals were obtained from the Department of Pathology, Escola Paulista de Medicina—Federal University of São Paulo, Brazil, following routine amputations. The control specimens were cleaned with a scalpel until all tissue in the cortical and medullary portions had been removed.

None of the bone specimens analysed arose from embalmed remains.

After washing in 0.9% saline solution, these specimens were placed in a 37 °C oven in sterile conditions for seven days prior to preparation of the donor blocks.

The specimens were sectioned and fixed in 14 ml 10% buffered formalin for 21 hours. After fixing, the sections were washed in water for 1 hour and decalcified using two different protocols. In protocol TMA-NA, 11 exhumed and 5 control bone sections were immersed in 15 ml 7% aqueous nitric acid. In protocol TMA-EA, 9 exhumed and 4 control bone sections were immersed in 15 ml 0.5 M EDTA solution in 0.5 M phosphate buffered saline. The decalcification agents were changed every two days until the samples were sufficiently decalcified for histological processing. The decalcified sections were washed in water for 10 min and inserted into plastic cassettes containing absolute alcohol prior to embedding in paraffin blocks. All bone specimens were sectioned transversely and embedded in blocks in the same orientation.

### Sample attributes

Each bone section was photographed and the following information recorded: period of burial (yr.), age at death (yr.), weight (g.), cortical surface color, medullary surface color, section dimensions (cm.) and decalcification time (days).

### Tissue microarray recipient block preparation

Two histological sections of 3 μm from each of the 20 exhumed and 9 control bone donor blocks were mounted on silanized slides and stained with hematoxylin and eosin (H&E) in order to permit selection of suitable areas from each block for subsampling. Cannulas with an oblique tip (Nipo Medical Ltda., Brazil) were used to subsample cores from each block. The length (50 mm) and larger inner diameter (1.74 mm) of these cannulas were found to be valuable attributes in subsampling cores from the compact femoral bone blocks. Two paraffin blocks, TMA-NA and TMA-EA, were used to receive cores subsampled from the donor blocks produced following 7% nitric acid and 0.5 M EDTA decalcification, respectively.

Core subsampling was performed in duplicate, with two adjacent cores being collected from each donor block and inserted into the receiver block with the aid of a needle of larger caliber. Special care was taken to ensure a horizontal distance of 1.5 mm and a vertical distance of 4 mm between each core. In addition to the 58 exhumed and control compact femoral bone cores, 2 specimens of hepatic tissue were added as positive controls for immunohistochemistry (see below). The two receiver blocks were placed in a 60 °C oven for 1 min. in order to ensure the inserted core had melted in to the receiver block. The receiver blocks were frozen for 25 min. immediately prior to histological sectioning and mounting onto silanized slides.

Block TMA-NA received cores from 11 exhumed and 5 control bone sections decalcified in 7% nitric acid as well as 2 further control cores from hepatic tissue, and 25 histological sections at 4 μm × 76 mm × 26 mm were obtained from this block. Block TMA-EA received cores from 9 exhumed and 4 control bone sections decalcified in 0.5 M EDTA as well as the 2 control cores from hepatic tissue, and 27 histological sections at 4 μm × 76 mm × 26 mm were obtained from this block.

H&E, periodic acid-Schiff (PAS), Silver Methenamine (SM) and immunohistochemical— anti CD31 and anti-CD34—staining was performed according to standard histopathology laboratory protocols (Department of Pathology, EPM-UNIFESP). Sample loss during TMA preparation was recorded as the percentage of subsample cores that could not be clearly observed in histological sections prepared from the multiplex.

### Image acquisition and analysis

Image analyses were performed using digital microscopy. ImageJ (National Institutes of Health, USA) was used for preliminary differentiation of structures and simultaneous analysis of images in series, prior to subsequent detailed image analysis.

### Statistical analysis

Statistica 12.5 (Dell Statistica, USA) was used in calculations of sample means and standard deviations applied to the exhumed bone and post-operative bone control samples, and the groups decalcified with 7% nitric acid and 0.5M EDTA, respectively. Comparisons of distributions were made for volumes, decalcification times, osteocytes per lacuna, percent bone matrix area loss, and fungal spheroid element ratios. The significance level was set at 0.05. Coefficients of correlation (R^2^) between decalcification time and specimen attributes for groups calcified with 7% nitric acid and 0.5M EDTA, respectively. Coefficients of correlation were also calculated and higher values—arbitrarily above 0.3—were chosen in order to examine relationships between period of burial and age at death, and osteocytes per lacuna, percent bone matrix area loss and fungal spheroid element ratios.

### Lacuna and osteocyte counts

The numbers of preserved lacunae and osteocytes were measured in images acquired from H&E stained slides at 400x magnification. The areas in each subsample with the highest numbers of osteocytes—‘hot-spots’ (see Fig. 1b)—were located and digitized in 3 consecutive fields per subsample in a zigzag pattern in both duplicates. Total lacuna and osteocyte counts in the 6 resultant fields were recorded using the Cell Counter Type I and Type II plugins in ImageJ.

### Percent bone matrix area loss

Bone matrix area loss was estimated in images acquired from H&E stained slides at 100x magnification. At this magnification, each subsample in the TMA could be completely visualized. The area of bone loss was highlighted using the Freehand Selections tool in ImageJ (see Fig. 1c and d) and deducted from the constant total area of each image in pixels to generate the percent bone matrix loss area per specimen in pixels. Ill-defined bone structures and artifacts were disregarded.

### Fungal spheroid element ratios

Measurement of fungal spheroid elements was performed semi-quantitatively in images acquired from PAS and SM stained slides at 100x magnification (see Fig. 1e and f). Evaluation was carried out by scoring the number Haversian systems affected by fungal spheroid elements to generate a fungal spheroid element ratio for each staining method. The highest score assessed in both duplicates was recorded.

### Detection of preserved endothelial cells

The presence of endothelial cells in the bone samples was qualitatively determined by analyzing slides stained by immunohistochemistry using the streptavidin-biotin-peroxidase standard technique^64^,^65^ with modifications, using anti-CD31 (DAKO, USA) and anti-CD34 (Dako, USA) antibodies considered to be present in samples with positive staining for anti-CD31 or anti-PECAM-1, and anti-CD34. Analyzes were performed at 200x magnification for the best representation (see Fig. 1g and h).

### Detection of preserved bone collagen fibers

The analysis of preservation of bone collagen and its architecture was qualitatively performed in slides stained with Picro-sirius red at 100x magnification using polarized light. Arrangements of thick and thin bundles of bone collagen were noted (see Fig. 1i and j), with regard to the known prevalence of type I collagen in cortical regions and type III collagen in medullary regions^66^.

## Additional Information

**How to cite this article**: Mello, R. B. *et al*. Tissue Microarray Analysis Applied to Bone Diagenesis. *Sci. Rep.* **7**, 39987; doi: 10.1038/srep39987 (2017).

**Publisher's note:** Springer Nature remains neutral with regard to jurisdictional claims in published maps and institutional affiliations.

## Figures and Tables

**Table 1:** 

Specimen	Period of Burial (yr.)	Age at Death (yr.)	Cortical Surface Color	Medullary Surface Color	Length (cm.)	Width (cm.)	Height (cm.)	Weight (g.)	Decalcification Time (days)	Lacuna Count	Osteocyte Count	Bone Matrix Area Loss (%)	PAS Fungal Element Ratio	SM Fungal Element Ratio	Combined Fungal Element Ratio
(**a**)															
Exhumed Bone															
004	15	—	Light brown	Red-brown	1.0	0.6	0.4	1.26	4	41	5	10.89	0.90	0.80	0.85
005	12	36	Light brown	Dark brown	1.0	0.5	0.4	0.17	4	78	10	4.30	0.45	0.40	0.43
006	12	67	Black	Dark brown	1.3	0.5	0.4	0.67	7	72	13	2.39	0.79	0.88	0.83
008	10	75	Red-brown	—	1.2	0.5	0.5	0.65	4	59	4	4.17	0.91	1.00	0.96
009	10	49	Light brown	Black	1.0	0.7	0.4	1.23	11	43	7	6.38	0.53	0.67	0.59
010A	10	27	Dark brown	—	0.9	0.4	0.3	0.19	4	111	22	4.15	1.00	1.00	1.00
010B	10	27	Dark brown	—	1.0	0.4	0.4	0.18	4	12	0	3.11	1.00	1.00	1.00
011	9	49	Dark brown	Black	0.8	0.4	0.3	0.48	4	19	1	11.74	1.00	1.00	1.00
012	9	49	Red-brown	Dark brown	0.7	0.5	0.4	0.47	4	37	5	7.38	1.00	—	1.00
014	9	51	Light brown	Dark brown	1.7	0.4	0.4	1.36	7	74	10	6.97	0.62	0.40	0.52
015	6	69	Dark brown	Black	0.6	0.5	0.4	0.41	8	81	9	2.87	0.78	0.73	0.75
**Mean**	**10.18**	**49.90**			**1.02**	**0.49**	**0.39**	**0.64**	**5.55**	**57.00**	**7.82**	**5.85**	**0.82**	**0.79**	**0.81**
**SD**	**2.27**	**16.78**			**0.30**	**0.09**	**0.05**	**0.45**	**2.38**	**29.62**	**6.15**	**3.16**	**0.20**	**0.24**	**0.21**
Control Bone															
16					1.0	0.5	0.5		13	90	25	0.31	0.00	0.00	0.00
17					1.0	0.2	0.2		12	71	20	0.25	0.00	0.00	0.00
18					1.0	0.3	0.3		13	116	27	0.80	0.00	0.00	0.00
19					1.0	0.3	0.3		13	118	28	0.93	0.00	0.00	0.00
21					1.3	0.2	0.1		12	149	36	0.89	0.00	0.00	0.00
**Mean**					**1.06**	**0.30**	**0.28**		**12.60**	**108.80**	**27.20**	**0.64**			
**SD**					**0.13**	**0.12**	**0.15**		**0.55**	**29.73**	**5.81**	**0.33**			
(**b**)															
Exhumed Bone															
013	9	—	Black	Red-brown	1.0	0.4	0.4	0.78	31	73	5	1.17	1.00	1.00	1.00
024	10.5	43	Red-brown	Light brown	1.0	0.4	0.3	0.31	47	180	9	0.74	0.00	0.00	0.00
025	10.4	60	Light brown	Light brown	1.2	0.4	0.3	0.24	52	128	3	2.02	1.00	1.00	1.00
026	10.2	61	Red-brown	Light brown	1.0	0.4	0.3	0.51	46	91	11	6.07	1.00	1.00	1.00
027	10.1	52	Light brown	Light brown	1.3	0.4	0.4	0.50	53	135	1	0.95	0.71	1.00	0.85
028	9.6	22	Light brown	Light brown	1.6	0.4	0.4	0.74	54	227	9	1.15	0.25	0.50	0.39
029	9.3	54	Light brown	Light brown	1.0	0.4	0.4	0.48	40	123	7	16.25	1.00	1.00	1.00
030	8.3	51	Black	Red-brown	1.0	0.4	0.4	0.57	25	308	13	24.90	1.00	1.00	1.00
031	8	58	Light brown	Light brown	0.9	0.3	0.3	0.13	38	55	1	0.91	0.86	0.90	0.88
**Mean**	**9.49**	**50.13**			**1.11**	**0.39**	**0.36**	**0.47**	**42.59**	**146.67**	**6.56**	**6.02**	**0.76**	**0.82**	**0.79**
**SD**	**0.91**	**12.76**			**0.22**	**0.03**	**0.05**	**0.22**	**10.18**	**80.29**	**4.33**	**0.44**	**0.38**	**0.35**	**0.36**
Control Bone															
32					1.5	0.3	0.3		59	68	17	0.47	0.00	0.00	0.00
33					1.4	0.4	0.4		57	88	13	0.49	0.00	0.00	0.00
34					1.5	0.3	0.4		61	81	15	0.41	0.00	0.00	0.00
35					0.7	0.3	0.4		55	81	36	0.41	0.00	0.00	0.00
**Mean**					**1.28**	**0.33**	**0.38**		**58.00**	**79.50**	**20.25**	**0.44**			
**SD**					**0.39**	**0.05**	**0.05**		**2.58**	**8.35**	**10.63**	**0.04**			

(a) Specimen attributes and quantitative results of histological analysis of 7% nitric acid decalcified group. (b) Specimen attributes and quantitative results of histological analysis of 0.5 M EDTA decalcified group.

**Table 2:** Means of specimen attributes and histological variables by decalcification group.

Variable	7% Nitric Acid Decalcified Group		0.5 M EDTA Decalcified Group	
	Exhumed Bone *n* = 11	Control Bone *n* = 5	Exhumed Bone *n* = 9	Control Bone *n* = 4
Volume (cm^3^)	0.22 ± 0.09	0.10 ± 0.09	0.16 ± 0.05	0.17 ± 0.06
Decalcification Time (days)	5.55 ± 2.38	12.6 ± 0.55	42.9 ± 10.18	58 ± 2.58
Osteocyte count	7.82 ± 6.15	27.20 ± 5.81	7.25 ± 4.06	20.25 ± 10.63
Bone matrix area loss (%)	5.85 ± 3.17	0.64 ± 0.33	6.02 ± 8.69	0.44 ± 0.04
PAS Fungal Element Ratio	0.82 ± 0.20	0.00	0.76 ± 0.38	0.00
SM Fungal Element Ratio	0.79 ± 0.24	0.00	0.82 ± 0.35	0.00
Combined Fungal Element Ratio	0.81 ± 0.21	0.00	0.79 ± 0.36	0.00

**Table 3:** Coefficients of correlation (R^2^) of decalcification time with specimen attributes.

Specimen Attribute	Coeffcient of Correlation (R^2^)	
	7% Nitric Acid Decalcified Group	0.5 M EDTA Decalcified Group
Period of Burial	0.0884	0.5893
Age at Death	0.1497	0.1497
Volume	0.1386	0.1292
Weight	0.2593	0.0332
Density	0.0473	0.3155
Length	0.0229	0.4476
Area of Largest Side	0.2010	0.3921
Surface Area	0.1311	0.1940

**Table 4:** Matrix of Coefficients of correlation (R^2^) between with specimen attributes and histological variables (7% nitric acid decalcified group is in bold text).

Variable	Coeffcient of Correlation (R^2^)					
	Period of Burial	Age at Death	Length	Osteocytes per Lacuna	Percent Bone Area Loss	Fungal Spheriod Element Ratio
Period of Burial			**0.0815**	**0.0000**	**0.0663**	**0.006**
Age at Death				**0.3235**	**0.0091**	**0.005**
Length	0.1306				**0.0081**	**0.1576**
Osteocytes per Lacuna	0.0894	0.3474			**0.0164**	**0.1082**
Percent Bone Area Loss	0.1813	0.0307	0.0953	0.0254		**0.0128**
Fungal Spheriod Element Ratio	0.1472	0.4840	0.0931	0.0152	0.1512	
